# DOES NEIGHBORHOOD GENTRIFICATION AFFECT FOOD INSECURITY AMONG URBAN OLDER ADULTS? EVIDENCE FROM NEW YORK CITY

**DOI:** 10.1093/geroni/igad104.2372

**Published:** 2023-12-21

**Authors:** Ethan Siu Leung Cheung

**Affiliations:** University of Utah, Salt Lake City, Utah, United States

## Abstract

Emerging studies have established that food insecurity is a prevalent public health problem among older adults, including those in urban areas. However, the relationship between neighborhood gentrification, an increasing trend in urban development, and food insecurity remains unclear. Using cross-sectional data from the Poverty Tracker Study, this study aimed to answer two research questions: 1) what is the association between living in gentrifying neighborhoods and food insecurity? 2) Do the food environment, public transportation, and social cohesion mediate the association? The sample consisted of 710 New York City residents aged 65 or older. Based on respondents’ census tracts, individual-level data were merged with neighborhood data obtained from the American Community Survey. local statistics were estimated by GeoDa 1.20 to examine the spatial distribution and significant clusters of generifying neighborhoods across New York City. ArcGIS 10.1 was then used to produce maps. To address both research questions, logistic regressions and mediation analyses were conducted. Regressions revealed that living in generifying neighborhoods was significantly associated with a greater risk of food insecurity, even after adjusting for demographic characteristics and socioeconomic status, such as income. Additionally, mediation analyses suggested that the association was significantly mediated by community social cohesion and the number of healthy food outlets. Findings suggest that neighborhood gentrification presents a salient risk factor for late-life food insecurity in urban areas. Community programs that foster social connections among neighbors and local food assistance programs may be particularly needed in gentrifying neighborhoods to mitigate and reduce the risk of late-life food insecurity.

